# Nurses in double trouble: Antecedents of job burnout in nursing profession

**DOI:** 10.12669/pjms.35.4.600

**Published:** 2019

**Authors:** Mirza Naveed Shahzad, Mirza Ashfaq Ahmed, Bushra Akram

**Affiliations:** 1 Dr. Mirza Naveed Shahzad, Assistant Professor, Department of Statistics, University of Gujrat, Pakistan; 2 Dr. Mirza Ashfaq Ahmed, Assistant Professor, Department of Management Sciences, University of Gujrat, Pakistan; 3 Dr. Bushra Akram, Assistant Professor, Department of Psychology, University of Gujrat, Pakistan

**Keywords:** Family support, Job burnout, Nursing profession, Working environment

## Abstract

**Objective:**

To understand the most prominent factors contributing to job burnout in the nursing profession.

**Methods:**

Mixed method design was used in this study. In the qualitative part of the study, a focus group discussion approach was used to determine the major factors contributing in nurses’ job burnout. The quantitative part was conducted by using a questionnaire based on the theme generated in the qualitative part along with other demographic information. The data was collected from 93 nurses with 90.3% response rate.

**Results:**

The proposed logistic regression model was able to correctly classify the 96% job burnout cases using factors mutually agreed in the focus group discussion. All the factors are significantly contributing to job burnout. However, the unfavourable work environment contributes more to job burnout as compared to the unfavourable support from family.

**Conclusion:**

unfavourable support of work environment and unfavourable support from family are the main contributors in the job burnout of nurses. Therefore, an equal improvements in both areas should be made on the priority basis to retain the happy nurses to deliver excellent healthcare services.

## INTRODUCTION

Superior performance is directly and strongly linked to the psychological well-being of the employees. Happy and satisfied employees remain engaged and put in sincere efforts to reach the highest standards of performance. However, in the healthcare services, the employees’ performance becomes more challenging as the nature of the job requires a high level of sensitivity and utmost accuracy where a small mistake can lead to severe consequences.^1^ In nursing profession, job performance is even more crucial because the patients’ recovery and safety depend mainly on the level of care and services provided by the nurses.^2^ Therefore, job satisfaction and engagement become the key points of discussion in the recent literature of nursing profession because nursing is the spine of healthcare services. Special emphasis is put on the enhancement of the psychological well-being of the nurses for the speedy recovery of the patients. Despite the sensitivity of the profession and all efforts made, the increasing rate of job stress and burnout has become a global challenge.^3^ This is mainly due to the unique nature of the nursing profession where work schedules, heavy workloads, peers’ behaviour, environmental factors, patient support, organizational characteristics, nature of diseases and family pressure directly influence the job performance.^4^,^5^ Therefore, the nurses, as an employees and a family members, are pressurized to justify both roles simultaneously. These mutually incompatible roles along with inconsistent and behavioural demands contribute immensely to the job burnout rate.^6^,^7^ Therefore, job burnout leads to the shortage of nurses all over the world and creates a huge gap between the needed and the available nurses to cater to the demands of the healthcare services.^8^

The shortage of qualified and well-trained work-force of the healthcare services in Pakistan has been highlighted at various forums to address this serious matter.^9^-^11^ Another very important aspect associated with this shortage is the reluctance of females to join this profession and at the same time, the growing tendency to leave this profession due to the aforementioned factors. Considering the facts described, the purpose of this study was to understand the problems associated with the nursing profession and to find out the most prominent factors contributing to the job burnout in the nursing profession.

## METHODS

This study was conducted in two stages with mixed method design. At the first stage qualitative approach was used in which one focus group was conducted to deeply understand the perception of the nursing profession in Pakistan and to determine the major contributors of job burnout associated with this profession. For this purpose, nine nurses were selected. The inclusion criterion was the work experience of at least 10 years, working both in morning and night shifts and in at least five different departments. The researcher coordinated the focus group discussion and finalized the key problem areas related to the nursing profession by following the modified Delphi Method used in the healthcare sector. Firstly, initial coding was done and a total number of 29 statements were generated. In Round-2, the similar statements were grouped together and reduced to 21. In Round-3, seventeen initial themes reached consensus. In these seventeen themes, four were individual themes and the thirteen others were grouped together into two main factors. The main factors were ‘unfavourable support from family’ and ‘unfavourable support from the working environment’, with six and seven themes, respectively.

At the second stage of the study, a quantitative approach was used in which a questionnaire was developed on the basis of seventeen initial themes that were generated via the results of a focus group. The English language was used in both themes discussed in the Focus Group Discussion and questions extracted from themes. The questionnaire comprised two parts. Part 1 included the demographic information and Part 2 included the thirteen statements categorized into two factors. Four other questions, including choice of the job, the problem faced during the night shift, embarrassing feelings of the family due to nursing and satisfaction with current salary structure were also included in the questionnaire other than the two main factors considering their strong impact on job burnout in Pakistani context as indicated by literature review.^12^ The questionnaire was presented to the panel of five psychologists for establishing the content and construct validity of the items. The questionnaire was given to 103 nurses from the registered private hospitals operating in Gujrat city and offering multi-specialty services. The participants were recruited through proportionate probability sampling technique. The total number of nurses on the hospital’s payroll was treated as the research frame (the research frame consisted of 147 nurses). 103 nurses were included on the basis of proportional allocation (N=103, 70% of the population from each hospital). The inclusion criteria include, worked in both morning and night shifts, five years and above work experience, and worked at more than one hospital. Questionnaires were handed to nurses to register the responses themselves. The reliability of the factors and questionnaire was tested through Cronbach’s alpha.

All the considered factors were highly reliable,^13^ as the Cronbach’s alpha coefficient of the two factors was 0.916 and 0.871, respectively. The questionnaire was also proven reliable on the basis of the value obtained through Cronbach’s alpha coefficient of 0.849. With this, confirmatory factor analysis (CFA) was also employed and the analysis proved that the questions in the two considered factors were highly significant and those factors followed the recommended goodness of fit indices.^14^ for CFA, as reported in the Table-I. Further demographic detail of the sample is presented in the Table-II.

**Table I:** The Model fit indices Goodness of Fit values

Factors	chi/d.f	GFI	AGFI	RMSEA
Model fit indices	<3	>0.90	>0.90	<0.08
Unfavourable support from family	0.162	0.926	0.934	0.047
Unfavourable support from working environment	1.827	0.918	0.907	0.069

Chi/d.f: Ratio of Chi-Square with Degree of Freedom, GFI: Goodness of Fit Index, AGFI: Adjusted Goodness of Fit Index, RMSEA: Root Mean Square Error of Approximation

**Table II:** Demographic characteristics of the respondents.

Characteristics	Category	N	%	Characteristics	Category	n	%
Marital Status	Single	83	89.2	Father’s Profession	Farmer	16	17.2
	Married	8	8.6		Labor	30	32.3
	Divorced	2	2.2		Business	20	21.5
Age (Years)	Below 20	6	6.6		Teaching	8	8.6
	21-25	60	65.9		Free at home	9	9.7
	26-35	20	22.0		Shopkeeper	5	5.4
	36-40	1	1.1		Late	5	5.4
	Above 40	3	3.3	Mother’s Profession	housewife	86	92.5
Salaries	below 10000	6	6.5		Heath worker	5	5.4
	10001-15000	52	55.9		Late	2	2.2
	15001-20000	22	23.7	Duty in Ward	No	21	22.6
	20001 and above	13	14.0		Yes	72	77.4

Total Number of respondents: 93

All of the research ethical standards were followed in the course of the study. The Questionnaire was withdrawn in case of an emergency and another meeting was scheduled. Utmost efforts were made to avoid the respondents suffering from extreme tiredness, long working hours, and an unpleasant situation caused by their interaction with patients, visitors or hospital management. Similarly, respondents were not persuaded in case of refusal to participate in the study. Consequently, 93 nurses completed the provided questionnaire with 90.3% response rate. Each questionnaire was assigned a unique code for traceability and entered in the SPSS for analysis purpose. The entered data was screened before applying the statistical analysis.

## RESULTS

Mixed Method design was used and a questionnaire was developed on the basis of the themes finalized in the focus group discussion. Using the questionnaire 103 nurses were interviewed in which ten questionnaires were discarded from the analysis due to incomplete information. The initial analysis is listed in Table-II, it reflects that more than 80% respondents were above 21 year of age and only 9% were married. To estimate the risk factors for job burnout potential confounders were entered into the logistic regression model including age, job by selection (by choice vs. by force), problem in night shift, family feel embarrassment, satisfaction with salary, unfavorable support from family and unfavorable support from working environment. All the considered risk factors were statistically significant (p-value ≤ 0.05) in the final logistic regression model. The odd ratios with other results of the model are presented in Table-III.

**Table III:** Logistic Regression for Nurses Job Burnout.

	β	SE(β)	Wald	p-value	OR	95% C. I	

						Lower	Upper
Age (X_1_)	0.2080	0.0510	16.6336	0.0000	1.2312	1.1141	1.3606
Job by choice (X_2_)	-0.958	0.173	30.6647	0.0005	0.3837	0.2733	0.5385
Problem in night shift (X_3_)	1.363	0.411	10.9979	0.0142	3.9079	1.7462	8.7457
Family feel embarrassment (X_4_)	1.815	0.226	64.4965	0.0136	6.1411	3.9434	9.5636
Satisfaction with salary (X_5_)	-0.469	0.112	17.5352	0.0163	0.6256	0.5023	0.7792
US from family (X_6_)	1.364	0.517	6.9606	0.0000	3.9118	1.4200	10.776
US from working environment (X_7_)	1.685	0.431	15.2843	0.0371	5.3925	2.3169	12.550
Constant	-1.609	1.867	0.7427	0.0000			

*Note:* Tests are two-tailed, Confidence Interval (C.I), Odd ratio (OR), Unfavourable support (US), age <30=0, >30=1, Job joined by choice No =0, Yes=1, Face problem in night shift No =0, Yes=1, Family members feel embarrassment (due to my nursing job) No =0, Yes=1, Satisfaction with salary No =0, Yes=1.

Odd ratio of age indicates, age after 30 year creates 23% more significant risk to get job burnout as compared to below 30 years. The respondents who have opted nursing profession by force are more likely to have job burnout than those who opted by choice. The value of the odd ratio for this risk factor is 0.3837 with negative coefficient (-0.958), that reveals, if nursing profession was selected by choice then it will decrease the job burnout. Similarly, ‘satisfaction with salary’ is protective against job burnout. The odd ratios of problems during night shift and family feeling embarrassed due to the nursing job are 1.363 and 1.815 with positive coefficients 3.9079 and 6.1411, respectively, therefore the presence of these risk factors, chances of job burnout in a nurse is increased by the odd ratio times.

The factors ‘unfavorable support from family’ and ‘unfavourable support of the working environment’ have positive association in developing job burnout as the odd ratios show that the nurses who have the ‘unfavorable support from family’ have 3.9118 times more risk of getting job burnout as compared to those who have favorable family support. A similar interpretation is made for the ‘unfavourable support of the working environment’, however, its impact is relatively higher in job burnout. Finally, the combine effect of all considered factors indicates that model is adequately fit and classified the cases with 96% accuracy.

## DISCUSSION

In Pakistan, nurses are mostly young, in early stage of their careers and agreed to work on low wage rate. However, on the other side, the experienced nurses were dissatisfied with their current salary growth and are inflexible to adapt themselves as per the requirements of business-oriented hospitals. Similarly, when age of the nurses corss the thirty years and they are not in this profession by choice then the chance of job burnout increases many times. This issue was loudly raised in the focus group discussion. Young age and profession opted unwillingly create another set of problems for nurses to perform their duties at night. The male dominant society and the social set-up do not encourage the females to stay out and perform their jobs at night. Especially for young and unmarried nurses, night shifts are highly undesirable as they feel more vulnerable to harassment. Similarly, night shifts are considered a nightmare by nurses having kids as it directly affects their family.^15^

Gender-based earning difference also exists in the local context and females are discriminated on earning and job status basis as compared to males.^16^ Social Closure Theory argues that males have a better chance of getting good job positions and higher salaries whereas similar opportunities are unavailable to females.^17^ Therefore, the salary based discrimination increases the chance of job burnout. Respondents of the study believe that earning plays an important role in the longevity of service as their satisfaction with earning has negative associations with job burnout.^18^ In the later stages of their career, they face more financial pressure to survive with less salary due to increased family demands and a higher rate of inflation. Therefore, with the passage of time, the financial pressure becomes the key contributor to bad health and job stress for nurses.

Females are unable to perform their job effectively without the support of their families. Work-family conflict is more obvious in females as compared to males.^19^ because females have to manage the family duties along with their work requirements. In the healthcare services, the job requires more concentration, higher level of accuracy, empathetic behaviour and alertness. Theory of Role Strain argues that each role being performed by individual demands a specific set of psychological, emotional and physical energy resources. However, the individuals are low on such energy resources. Allocation of resources to one role minimizes the same resources available for another role. Therefore, nurses are stuck between maintaining the full-time employment and meeting the caring and financial demands of their families. Nurses are exhausted due to depletion of available resources after performing full-time duties and need rest and psychological support from family to gain energy for other tasks associated with their families. However, failure in gaining the favourable support from the family results in the stressful impact on the work-family role balance.^19^-^21^ Therefore, the favourable support from family plays a crucial role in the career length and psychological well-being in the nursing profession.^8^,^22^,^23^

In the nursing profession, positive work-environment results in fewer job-related injuries, fewer chances of job burnout and maintaining a higher level of job satisfaction. On the other end, the negative work-environment results in poor outcomes associated with patients such as increased complication and mortality rate, prolonged treatment and increased treatment costs.^24^ Furthermore, a good work-environment facilitates to develop an excellent nurses’ relation, a supportive style of management, a well-coordinated aura, balanced workload and schedule, a well-established skill transference system and maintained psychosocial well-being of nurses.^4^,^5^ However, in the developing countries, nursing work-environment has been reported as less supportive, stressful, unfriendly, having an imbalanced work schedule and disrespectful. Such work-environment leads to job burnout, low retention rate, absenteeism, low job engagement, and productivity.^22^,^23^ Unfavourable work-environment is considered as the main cause of job burnout in the healthcare sector of Pakistan.^24^ The chance of job burnout, due to the unfavourable work-environment, is reported five times (5.3925) higher than the nurses working in the favourable working environment.

## CONCLUSION

Favourable support from both family and work environment must be provided to allow the nurses to have long and satisfied job career because provision of quality healthcare services is based on the stable and satisfied job career. However, in Pakistan, unfortunately, unfavourable support from the organization as well as family significantly contribute in the job burnout. Therefore, improvements in these areas should be addressed on the priority basis to retain the happy nurses to provide better patients’ care. Furthermore, special efforts should be made to attract talented students and higher social classes in the nursing profession to minimize the families’ embarrassment associated with the nursing profession.
